# Simple Synthesis of CeO_2_ Nanoparticle Composites *In Situ* Grown on Carbon Nanotubes for Phenol Detection

**DOI:** 10.3389/fchem.2022.907777

**Published:** 2022-05-17

**Authors:** Chao Hu, Haiping Huang, Yu Yan, Yongmei Hu, Sui-Jun Liu, He-Rui Wen

**Affiliations:** ^1^ Jiangxi Provincial Key Laboratory of Functional Molecular Materials Chemistry, School of Chemistry and Chemical Engineering, Jiangxi University of Science and Technology, Ganzhou, China; ^2^ Key Laboratory of Testing and Tracing of Rare Earth Products for State Market Regulation, Jiangxi University of Science and Technology, Ganzhou, China

**Keywords:** carbon nanotube, phenol, electrochemical sensor, nanocomposites, cerium dioxide

## Abstract

*via* simple hydrothermal method, CeO_2_ was *in-situ* grown onto the CNTs to form CeO_2_/CNTs nanocomposites were synthesized with cerium nitrate as Ce resource. The morphology and structure were characterized by transmission electron microscopy and X-ray diffraction. The characterizations reveal that CeO_2_ nanoparticles are uniformly dispersed onto the surface of the pre-acidified CNTs. The electrochemical property of the synthesized nanocomposite was investigated in 0.1 M KCl electrolyte containing 2 mM [Fe(CN)_6_]^3-/4-^. The nanocomposites were employed to fabricate electrochemical sensor for phenol detection. The linear range for phenol detection measured by the differential pulse voltammetry method is 1–500 μM. The sensor also exhibits good selectivity, reproducibility and stability. When applied for the river and tap water analysis, it shows good recovery rate.

## Introduction

At present, human life is inseparable from chemical products. As the chemical industry brings great convenience to our daily lives, it also damages our environment, making the global water pollution problem more and more serious (Lopez-Pacheco et al., 2019; Liu J. et al., 2021). Phenol is such a common pollutant in the chemical industrial wastewater, which can cause pollution to water bodies and the atmosphere, and also has strong chemical toxicity to human beings (Gruzdev et al., 2015; Singh and Chandra, 2019; Wang and Chen, 2020). Excessive exposure to water containing phenol can cause damage to the skin and eyes, and it also causes nerve damage and increases the risk of cancer (Singh and Chandra, 2019). Not only the World Health Organization (WHO) lists it as the third category of carcinogens, the European Union (EU) and the US Environmental Protection Agency also list it as an important environmental pollutant (Diaz-Gonzalez et al., 2016). What’s more, because phenol is difficult to degrade in the natural environment, its environmental pollution will eventually destroy the ecology system. Therefore, it is urgent to develop a technology that can quickly detect the phenol content in river water. At present, the main methods for detecting phenol includes spectrophotometry, gas chromatography-mass spectrometry, liquid/solid phase extraction/microextraction, high-performance liquid chromatography, etc (Alcudia-Leon et al., 2011; Jaworek, 2018; Liu W. et al., 2021) However, the operations for these methods are relatively complex, and the instruments are expensive, which limit their rapid and wide detection. Compared with the previous analysis methods, electrochemical analysis has the advantages of good stability, high sensitivity, low cost, and easy operation (Curulli, 2020; Tajik et al., 2020). Owing to these advantages, it is widely employed for the electrochemical detection of inorganic ions, small organic molecules and bio-molecules, etc.

One key factor in improving the performance of electrochemical sensors is to find suitable materials for modifying working electrodes (Ferrier and Honeychurch, 2021). So far, scientists have done a lot of research on this, and many materials with excellent electrochemical properties have been used to improve the performance of sensors (Abbas and Amin, 2022; Shao et al., 2022). Among them, the carbon nanotube is treated as an ideal material for the chemical modified electrode owing to excellent performance (Zhang and Du, 2020; Billing, 2021). Carbon nanotubes are composed of pure carbon atoms that interact through strong sp^2^ carbon-carbon bonds. They exhibit the unique carbon network geometry of tubular structures in nanoscale diameters and microscale lengths. The strong chemical bonds in the carbon network make CNTs the most fascinating nanomaterials. Because of the unique physical and chemical properties such as high mechanical strength, large surface area and electrical conductivity, it is widely utilized in the electrochemical fields such as electrochemical sensor (Wang J. et al., 2018), electrochemical catalyst (Tafete et al., 2022), supercapacitor (Yang et al., 2020), etc.

Due to the unique electronic configuration, rare earth elements are currently the hot-topic research materials (Huang and Zhu, 2019). Take Cerium (Ce) as an example, Ce is a member of the lanthanide family of metals, and it is the most abundant element of the rare earth metals found in the earth crust (Algethami et al., 2018). It is easy to lose outer electrons to form compounds of different valence states, thus making its chemical properties very active. The oxide of cerium, called ceria, is a rare earth semiconductor material with a low price and a wide range of applications. CeO_2_ has a cubic fluorite structure, in which the Ce element has two oxidation states Ce^4+^ and Ce^3+^. It is widely used in luminescent materials (Huang et al., 2021) catalysts (Zhu et al., 2019), electrode materials (Xiao et al., 2018; Huang et al., 2019), and so on. For the purpose of further exploring the electrochemical application of CeO_2_, in this study, a simple hydrothermal route was used to *in situ* grow CeO_2_ nanoparticles on the surface of carbon nanotubes. The synthesized CeO_2_/CNTs composites were employed to construct a phenol electrochemical sensor. The experimental results show that the CeO_2_/CNTs modified electrode has a good detection effect on phenol.

## Experimental

### Preparation of CeO_2_/CNTs

First, the CNTs were acidified with a mixed acid solution (V_98% concentrated sulfuric acid_: V_68% concentrated nitric acid_ = 3:1) at 90°C for 4 h under stirring and refluxing. Ce(NO_3_)_3_·6H_2_O was used as the cerium source to synthesize CeO_2_/CNTs composite material in one step by hydrothermal method. Dissolve 1.2 g Ce(NO_3_)_3_·6H_2_O and 0.1 g treated CNTs into 60 ml deionized water. After adjust the solution to pH = 9.0 with 0.5 M NaOH and stir for 1 h, it was then transferred into the autoclave and reacted at 160°C for 24 h. After that, it was allowed to cool naturally, and the product was centrifuged, washed, dried under vacuum, and ground. The similar route was used to prepare CeO_2_ nanoparticles without the addition of treated CNT at the beginning.

### Preparation of Electrochemical Sensor

The modified glassy carbon electrodes (GCE, *Φ* = 4 mm) of CeO_2_/CNTs/GCE, CeO_2_/GCE and CNTs/GCE were used as working electrode. All the cyclic voltammetric (CV) and differential pulse voltammetric (DPV) responses were recorded on electrochemical workstation.

Other detailed experimental procedures and apparatus parameters are provided in the Supplementary Material.

## Results and Discussion

### Material Characterization

**FIGURE 1:**
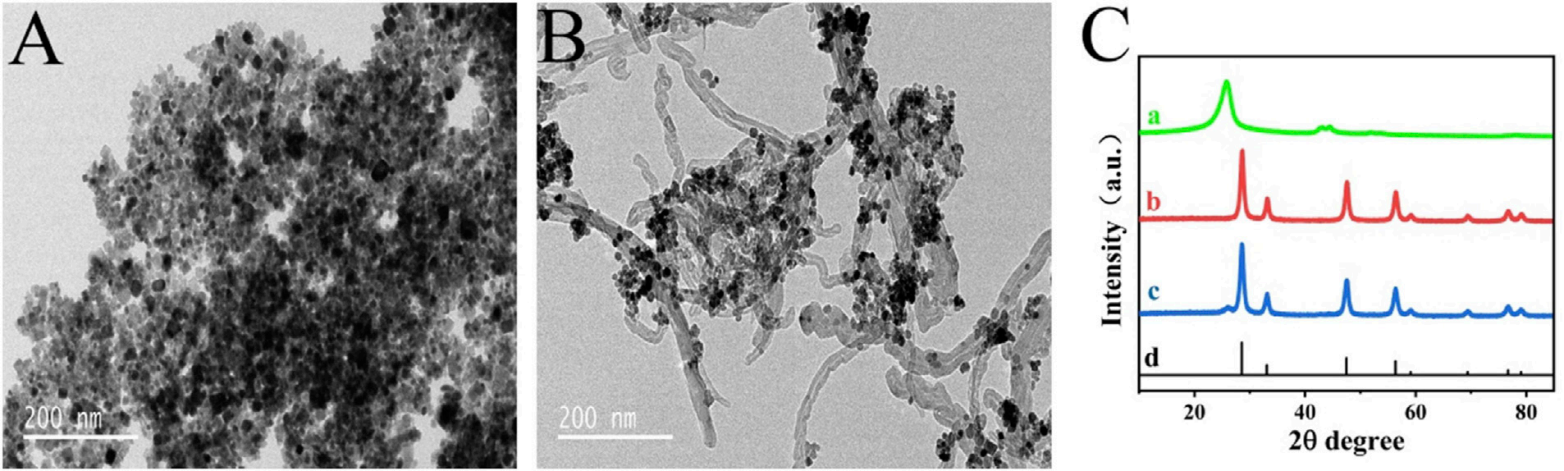
TEM images of CeO_2_ **(A)**, CeO_2_/CNTs **(B)**, and XRD patterns **(C)** of CNTs (a), CeO_2_ (b), CeO_2_/CNTs (c) and standard spectrum of CeO_2_ (d).

TEM and XRD technologies were employed for the purpose of intuitively observing the morphology and structure of the nanomaterials. Shown in Figures 1A,B is the TEM image of CeO_2_ nanoparticles and CeO_2_/CNTs nanocomposites. It can be seen that CeO_2_ nanoparticles are grown uniformly on the surface of CNTs. In the XRD spectra of Figure 1C, the diffraction peak at 26° in the curve 1) is the characteristic peak of CNTs. For CeO_2_, it can be seen from curve b in Figure 1C that a series of sharp diffraction peaks appear at 28.3°, 33.1°, 47.5°, 58.2°, which are correspond to the (111), (200), (220), (311) planes of CeO_2_ (Xiao et al., 2019). This is also consistent with the standard XRD spectrum of CeO_2_ (curve d, JCPDS card No. 34–0,394). And all these peaks are appeared in the CeO_2_/CNTs nanocomposite (curve c), proving the successful preparation of CeO_2_/CNTs composite.

### Cyclic Voltammetric Response of Different Electrodes

**FIGURE 2:**
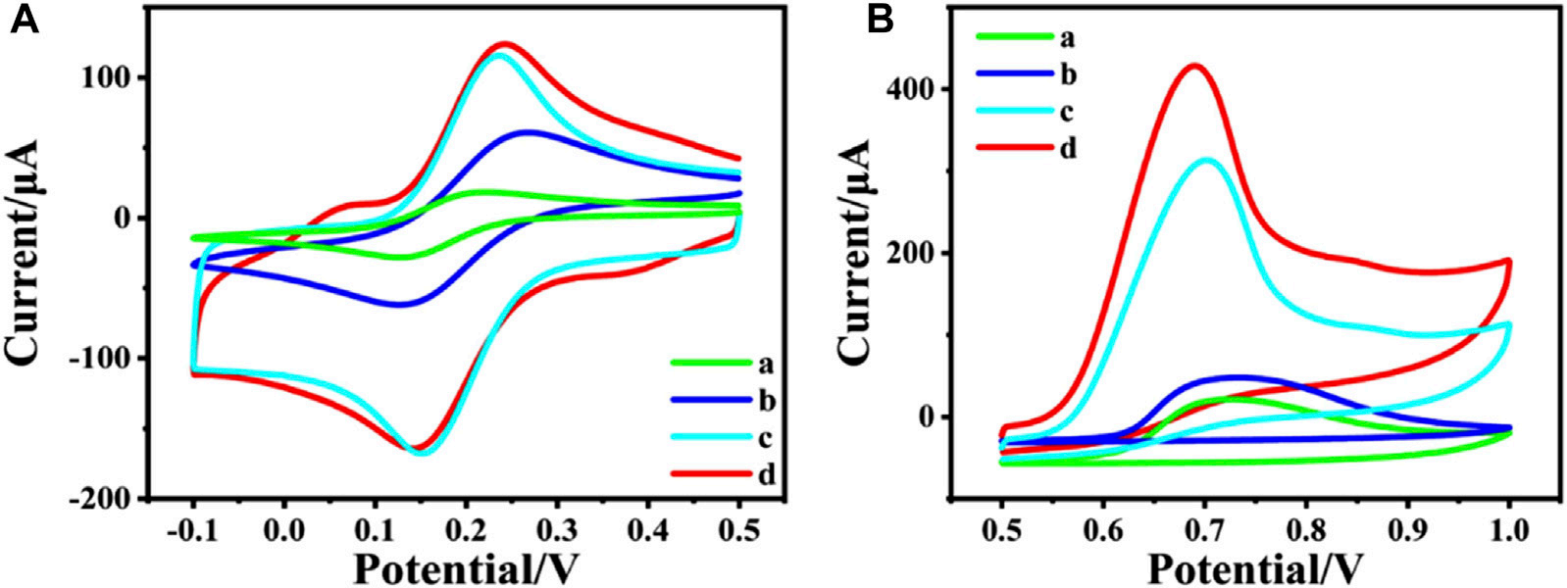
CVs of different electrode in 2 mM [Fe(CN)_6_]^3-/4-^ + 0.1 M KCl **(A)** and in phosphate buffer (pH = 5.0, 0.1 M) containing 2 mM phenol **(B)**. (a) bare GCE, (b) CeO_2_/GCE, (c) CNTs/GCE and (d) CeO_2_/CNTs/GCE. Scan rate: 50 mV s^−1^.

To study the electrochemical property of the nanocomposites, the CV responses of different nanocomposites modified electrode were recorded in 0.1 M KCl electrolyte containing 2 mM [Fe(CN)_6_]^3-/4-^, which are shown in Figure 2A. Compared with bare GCE (curve a), CeO_2_/GCE (curve b) shows a little bigger CV response. This confirms that as a rare earth semiconductor material, CeO_2_ can still promote the electron transfer between the electrode and the electrolyte. Nevertheless, the peak current is dramatically enlarged after CNTs are modified onto the GCE as CNTs/GCE (curve c), due to the excellent conductivity of CNTs. And the peak current is further enlarged for CeO_2_/CNTs/GCE (curve d). This proves that the binary composite of CeO_2_/CNTs owes the best electrochemical performance than single CNT or CeO_2_.

In order to investigate the electrochemical catalytic effect of the different nanomaterial towards oxidation of phenol, CV tests were performed on different modified electrodes in 0.1 M phosphate buffer containing 2 mM phenol in Figure 2B. It can be found that both CeO_2_/GCE (curve b in Figure 2B) and CNTs/GCE (curve c in Figure 2B) responses are better than the bare GCE (curve a in Figure 2B), which means both CeO_2_ nanoparticles and carbon nanotubes have a certain catalytic effect toward the electrochemical oxidation of phenol. And CeO_2_/CNTs/GCE (curve d in Figure 2B) has the largest oxidation peak compared to other modified electrodes. This shows that CeO_2_/CNTs nanomaterials have the best electrochemical catalytic effect on phenol. This is due to the synergistic catalysis effect between CeO_2_ nanoparticles and CNTs.

### Effect of Scan Rate

**FIGURE 3:**
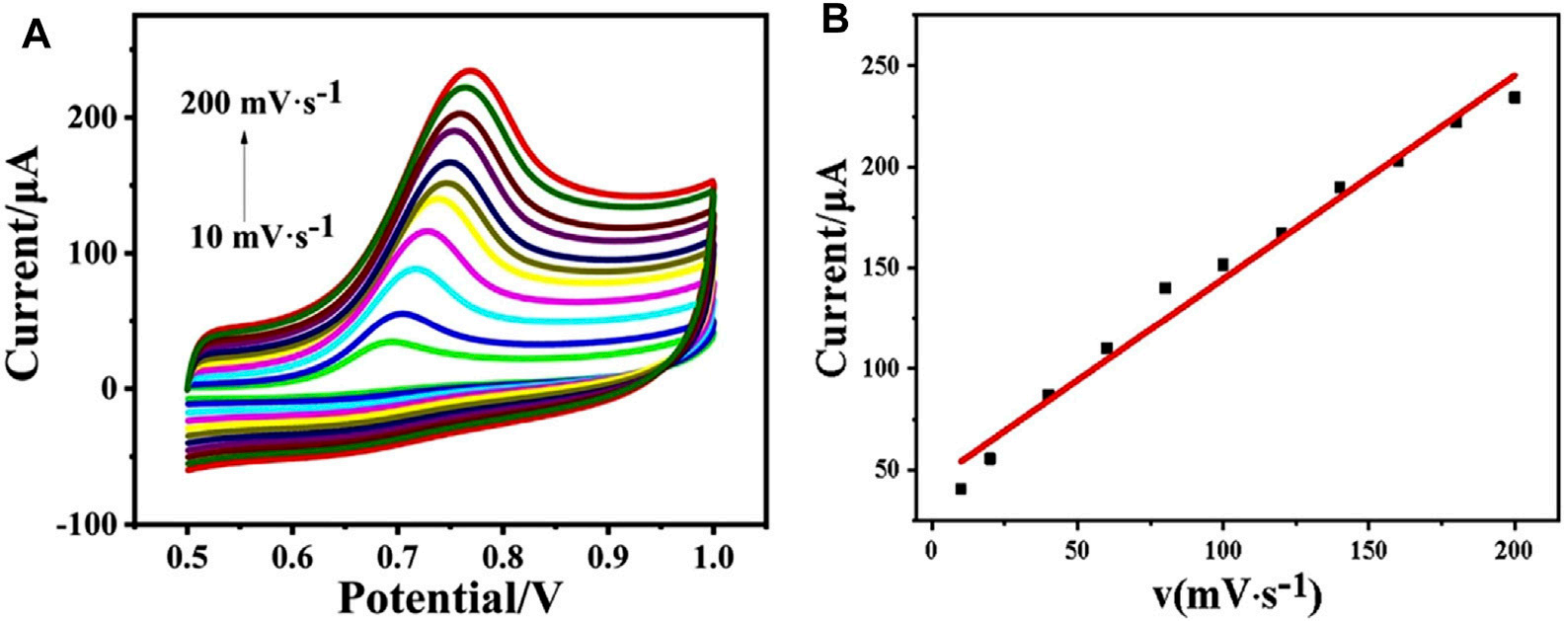
CVs of CeO_2_/CNTs/GCE in phosphate buffer (pH = 5.0, 0.1 M) with 1 mM phenol at different scan rates (10–200) mV·s^−1^ **(A)**, and the linear curve of the peak current *vs.* the scan rate **(B)**.

The electrochemical kinetic behavior of the as-prepared electrode was studied by CV in phosphate buffer (pH = 5.0, 0.1 M) containing 1 mM phenol (Figure 3). When the scan rate enlarges from 10 to 200 mV s^−1^, the oxidation peak current increases accordingly (Figure 3A). Figure 3B shows the linear curve of peak current value *vs*. the scan rate, where *I*_p_ (μA) = 44.00961 + 1.00644 *v* (mV·s^−1^) with *R* ^2^ = 0.98044. It confirms the kinetic behavior of CeO_2_/CNTs/GCE is a surface-controlled process.

### Effect of the pH Value

**FIGURE 4:**
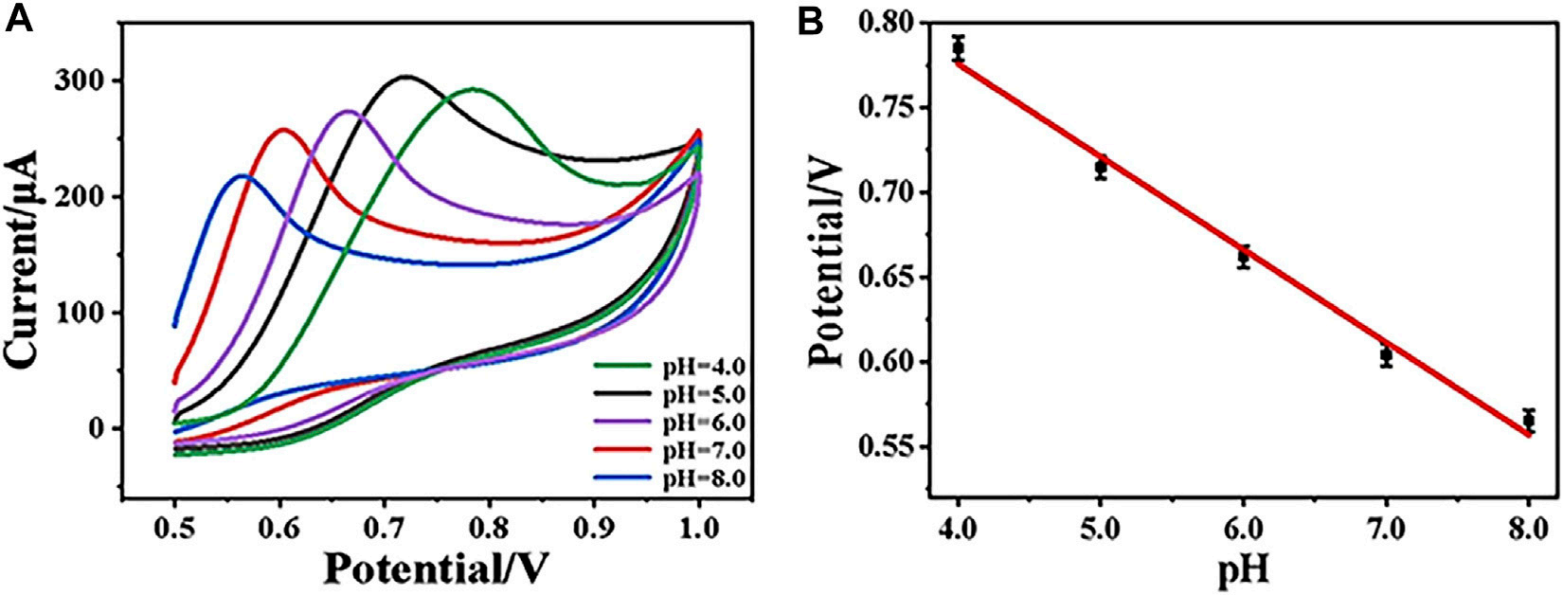
CVs of CeO_2_/CNTs/GCE in electrolyte with pH from 4.0 to 8.0 **(A)**, and the linear curve of peak potential *vs*. pH **(B)**.

During electrochemical analysis, the pH value of the solution plays an important role for the target determination. Herein, in order to study the effect of pH on the electrochemical performance of CeO_2_/CNTs/GCE, the CV behaviors of CeO_2_/CNTs/GCE in electrolytes with different pH values were recorded. As shown in Figure 4A, when the pH value increases from 4.0 to 8.0, the oxidation peak potential obviously shifts to the lower voltage direction. Figure 4B is the linear curve between the pH value of the solution and the oxidation peak potential of phenol, where *E*_p_(V) = 0.99462–0.05473pH with *R* ^2^ = 0.98843. Through calculation, the ratio value between the involved number of protons and electrons in the reaction is approximately 1. This is consistent with the transfer number of protons and electrons in the phenol oxidation reaction. At the same time, it is observed in Figure 4A that the electrode has the largest response current at pH = 5.0, so subsequent electrochemical experiments are carried out under the optimal pH value of 5.0.

### Determination of Phenol

**FIGURE 5:**
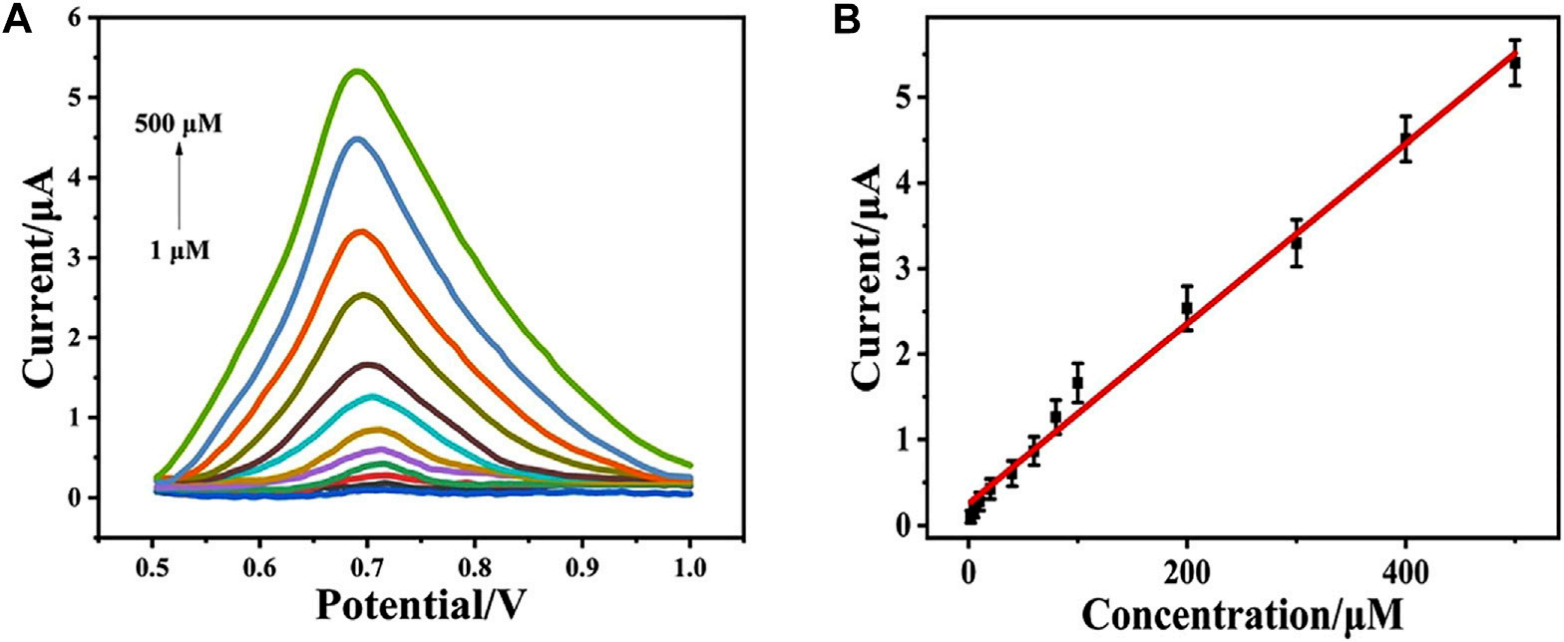
DPVs for CeO_2_/CNTs in phosphate buffer (pH = 5.0, 0.1 M) with different concentrations of phenol (1–500 μM) **(A)** and the corresponding plots of the oxidation peak currents at peak potentials *vs*. the concentrations of phenol **(B)**.

Under the optimal condition, the DPV response of CeO_2_/CNTs/GCE upon the addition of different concentration of phenol was recorded. Figure 5A is the DPV signals recorded in a phosphate buffer (pH = 5.0) with different phenol concentrations in the range of 1–500 μM. It can be seen from Figure 5A that as phenol concentration gradually increases, the corresponding oxidation peak current also increases. Drawn from the DPV curves, the linear equation (Figure 5B) between the DPV peak current (*I*_p_) and the phenol concentration (*c*_phenol_) is *I*_p_(μA) = 0.252 + 0.105 *c*_phenol_ (μM) with *R* ^2^ = 0.992. The detection limit for phenol is 0.3 μM. In contrast to other published electrochemical sensors, as shown in Table 1, CeO_2_/CNTs/GCE has a lower detection limit and a wide detection range. It shows that the sensor in this system exhibits satisfied performance.

**TABLE 1:** Comparison of different sensor performance.

Electrodes	Method	Linear Range (μM)	Detection Limit (μM)	Ref
Ni/MWCNT/GCE	CV	10–480	7.07	Yajing Wang et al. (2018)
Pt/g-C_3_N_4/_GCE	DPV	2–20	0.667	Song et al. (2019)
Na^+^-doped g-C_3_N_4_/CP	CV	1–110	0.23	Yin et al. (2020)
Fe_3_O_4_/AGO^a^/GCE	DPV	0.45–56, 156–456	0.4	Meng et al. (2019)
Fe_3_O_4_/MWCNT/GCE	DPV	5–235	4.83	Jiankang Wang et al. (2018)
CeO_2_/CNTs/GCE	DPV	1–500	0.3	This work

a AGO, for Amino-Functional Graphene.

### Selectivity and Reproducibility

**FIGURE 6:**
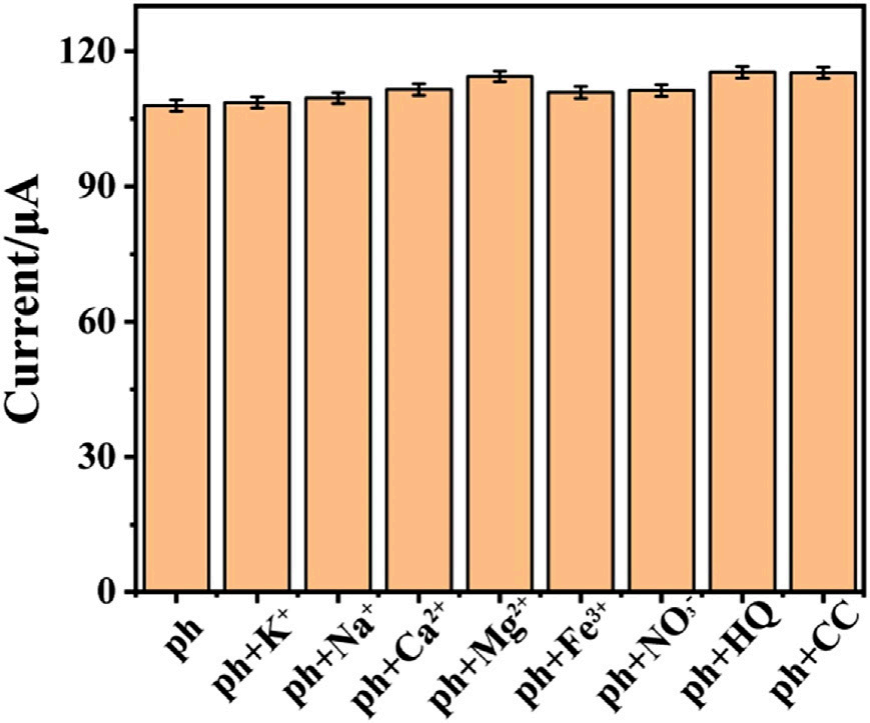
The selectivity study of CeO_2_/CNTs/GCE upon addition of different interferences.

In order to study the selectivity of the electrochemical sensor, other common substances are added into 0.1 M phosphate buffer (pH = 5.0) with 1 mM phenol, so as to record interfere effect for the electrochemical response of phenol. Based on the previous report about the anti-interference investigation for phenol detection, potassium chloride, calcium chloride, sodium chloride, iron chloride, sodium nitrate, magnesium chloride, hydroquinone (HQ) and catechol (CC) are chosen for anti-interference research. The result is shown in Figure 6. After adding different interferences (0.1 mM) into electrolyte, the electrochemical signal almost remains unchanged as compared to solo phenol detection. This illustrates that the prepared sensor has good selectivity. To investigate the repeatability of the sensor modification, five CeO_2_/CNTs modified electrodes were fabricated under the same conditions to measure 100 μM phenol solution. The calculated relative standard deviation (RSD) is 5.76%, which shows that the sensor has good reproducibility. And the stability is studied by measuring the CV response of the sensor in 100 μM phenol solution after stored in a desiccator for 1 week. Its signal value was 98.6% of the initial value.

### Real Sample Detection

DPV determinations of phenol in real samples of river and tap water were estimated using standard addition method to assess the possibility for real sample detection. Table 2 shows the actual sample detection results. The results show that the sensor in this system has good recovery rate and RSD in both river and tap water analysis. This proves that CeO_2_/CNTs/GCE is a reliable and effective platform for phenol detection in real sample.

**TABLE 2:** Real sample detection in river water and tap water.

**River Water**					
**Specimen**	**Concentration**	**Addition (μM)**	**Found (μM)**	**Recovery (%)**	**RSD (%)**
1	ND^a^	60.0	60.87	101.4	5.4
2	ND	100.0	101.28	101.28	6.5
3	ND	300.0	302.7	100.9	7.8
tap water					
4	ND	60.0	60.6	101.0	5.3
5	ND	100.0	101.2	101.2	6.1
6	ND	300.0	301.9	100.6	7.4

a ND, for Not Detected.

## Conclusion

CeO_2_/CNTs nanocomposites were synthesized by hydrothermal method. CeO_2_ nanoparticles are obtained with cerium nitrate as Ce resource. TEM images reveal that CeO_2_ nanoparticles are uniformly dispersed onto the surface of the pre-acidified CNTs. XRD spectra show that all the characteristic peaks of CNT and CeO_2_ are appeared in the CeO_2_/CNT nanocomposite. The CV responses in 0.1 M KCl electrolyte containing 2 mM [Fe(CN)_6_]^3-/4-^ prove that, as compared to the bare CNTs and CeO_2_, the CeO_2_/CNTs/GCE owes the best electrochemical performance. When applied for the electrochemical catalytic effect towards oxidation of phenol, CeO_2_/CNTs nanomaterials have the best catalytic effect on phenol oxidation. The CeO_2_/CNTs nanocomposites based electrochemical sensor displays wide linear range, good selectivity, reproducibility and stability, as well as the potential application for real sample detection.

## Data Availability

The original contributions presented in the study are included in the article/Supplementary Material, further inquiries can be directed to the corresponding author.
