# Supplementary material for: Simple Synthesis of CeO2 Nanoparticle Composites In Situ Grown on Carbon Nanotubes for Phenol Detection
Source: Front Chem. 2022 May 17;10:907777. doi: 10.3389/fchem.2022.907777 (PMC9152013; doi:10.3389/fchem.2022.907777)
Supplement: Supplementary file 1 [file DataSheet1.docx]

**Supplementary Material**

**Simple synthesis of CeO_2_ nanoparticle composites grown on carbon nanotubes in situ for phenol detection**

Chao Hu^1^, Haiping Huang^1,2*^, Yu Yan^1^, Yongmei Hu^2^, Sui-Jun Liu^1^ and He-Rui Wen^1^

^1^ Jiangxi Provincial Key Laboratory of Functional Molecular Materials Chemistry, School of Chemistry and Chemical Engineering, Jiangxi University of Science and Technology, Ganzhou 341000, P. R. China

^2^ Key Laboratory of Testing and Tracing of Rare Earth Products for State Market Regulation, Jiangxi University of Science and Technology, Ganzhou 341000, P. R. China

**1. Experimental**

**1.1 Materials and Apparatus**

CNTs were provided from XFNANO Materials Tech Co., Ltd (Nanjing China). Ce(NO_3_) _3_·6H_2_O purchased from Aladdin Chemical Reagent Co. Ltd (Shanghai, China). Phenol and other reagents were purchased in Sinopharm Chemical Reagent Co. Ltd (Shanghai, China). The XRD was characterized by PANalytical X’Pert Pro X-ray diffractometer with Cu Kα radiation (λ =0.15418 nm) at a scanning speed of 10º/min and diffraction angle between 10º and 85º. The transmission electron microscopy (TEM) images were obtained by FEI Tecnai G20. All electrochemical results were obtained on Chenhua electrochemical workstation (CHI 660D, Shanghai China). The conventional three-electrode system was employed with a modified glassy carbon electrode (GCE) as the working electrode, a saturated calomel electrode (SCE) as the reference electrode, and a Pt wire as the counter electrode.

**1.2 Preparation of modified electrodes**

The GCE (Φ=4 mm) was pre-cleaned on a polishing cloth with aluminum oxide powder (0.3 mm and 0.1 mm), then10 μL CeO_2_/CNTs suspension (5 mg/L in water) was dropped on the GCE surface and dried at room temperature. Finally, 3 μL chitosan solution (0.5 g in 100 mL 1% acetic acid) was used to seal CeO_2_/CNTs/GCE. The similar method was used to prepare CeO_2_/GCE and CNTs/GCE respectively.
